# Democratizing data-independent acquisition proteomics analysis on public cloud infrastructures via the Galaxy framework

**DOI:** 10.1093/gigascience/giac005

**Published:** 2022-02-15

**Authors:** Matthias Fahrner, Melanie Christine Föll, Björn Andreas Grüning, Matthias Bernt, Hannes Röst, Oliver Schilling

**Affiliations:** Institute for Surgical Pathology, Medical Center–University of Freiburg, Faculty of Medicine, University of Freiburg, Breisacher Straße 115a, D-79106 Freiburg, Germany; Faculty of Biology, Albert-Ludwigs-University Freiburg, Schänzlestraße 1, D-79104 Freiburg, Germany; Spemann Graduate School of Biology and Medicine (SGBM), University of Freiburg, Albertstraße 19A, D-79104, Germany; Institute for Surgical Pathology, Medical Center–University of Freiburg, Faculty of Medicine, University of Freiburg, Breisacher Straße 115a, D-79106 Freiburg, Germany; Khoury College of Computer Sciences, Northeastern University, 440 Huntington Ave, Boston, MA 02115, USA; Department of Computer Science, University of Freiburg, Georges-Köhler-Allee 106, D-79110 Freiburg, Germany; Young Investigators Group Bioinformatics and Transcriptomics, Helmholtz Centre for Environmental Research–UFZ, Permoserstraße 15, D-04318 Leipzig, Germany; Donnelly Centre, University of Toronto, 160 College St, Toronto, ON M5S 3E1, Canada; Institute for Surgical Pathology, Medical Center–University of Freiburg, Faculty of Medicine, University of Freiburg, Breisacher Straße 115a, D-79106 Freiburg, Germany; German Cancer Consortium (DKTK) and German Cancer Research Center (DKFZ), Hugstetter Straße 55, D-79106 Freiburg, Germany; BIOSS Centre for Biological Signaling Studies, University of Freiburg, Schänzlestraße 18, D-79104 Freiburg, Germany

**Keywords:** data-independent acquisition, proteomics, mass spectrometry, computational workflows, bioinformatics, galaxy

## Abstract

**Background:**

Data-independent acquisition (DIA) has become an important approach in global, mass spectrometric proteomic studies because it provides in-depth insights into the molecular variety of biological systems. However, DIA data analysis remains challenging owing to the high complexity and large data and sample size, which require specialized software and vast computing infrastructures. Most available open-source DIA software necessitates basic programming skills and covers only a fraction of a complete DIA data analysis. In consequence, DIA data analysis often requires usage of multiple software tools and compatibility thereof, severely limiting the usability and reproducibility.

**Findings:**

To overcome this hurdle, we have integrated a suite of open-source DIA tools in the Galaxy framework for reproducible and version-controlled data processing. The DIA suite includes OpenSwath, PyProphet, diapysef, and swath2stats. We have compiled functional Galaxy pipelines for DIA processing, which provide a web-based graphical user interface to these pre-installed and pre-configured tools for their use on freely accessible, powerful computational resources of the Galaxy framework. This approach also enables seamless sharing workflows with full configuration in addition to sharing raw data and results. We demonstrate the usability of an all-in-one DIA pipeline in Galaxy by the analysis of a spike-in case study dataset. Additionally, extensive training material is provided to further increase access for the proteomics community.

**Conclusion:**

The integration of an open-source DIA analysis suite in the web-based and user-friendly Galaxy framework in combination with extensive training material empowers a broad community of researches to perform reproducible and transparent DIA data analysis.

## Background

Data-independent acquisition (DIA) is a recently developed method addressing the need for reproducible and robust explorative proteomic measurements in larger sample cohorts [1]. Compared to classical data-dependent acquisition (DDA) measurements, in DIA all MS1 precursor peptide ions within a predefined *m*/*z* range (“window”) are fragmented and subjected to MS2 scans. Especially for high-throughput studies with dozens of samples, DIA has been shown to yield higher numbers of identifications and quantifications [2–4]. Furthermore, when compared to isobaric labeling approaches, DIA is less susceptible to batch effects stemming from chemical tagging and allows for quantitative proteome comparison in large cohorts [5,6]. Multiple DIA strategies have been developed over the past decade as reviewed in Ludwig et al. [3]. Applying overlapping isolation windows and subsequent demultiplexing has been shown to improve the precursor selectivity [7,8]. However, for DIA data with overlapping isolation windows, specific data processing may be required to ensure compatibility with subsequent data processing steps.

Different data processing strategies have been developed for DIA data, where the most common strategies apply a spectral library to enable the confident identification of peptides in DIA data [4]. Owing to the complex MS2 spectra as well as the requirement for a priori knowledge, a complete DIA data analysis can be divided into 3 steps: (i) the generation of a spectral library, (ii) the actual identification by matching measured fragment masses and their respective retention times (RTs) to the precursor and fragment information within the spectral library, and (iii) a statistical follow-up analysis yielding the identification of significantly altered protein expression profiles. A prototypical DIA analysis often includes a multitude of software and system environments for steps such as spectral library generation, peptide and protein identification in DIA measurements, and differential statistics. Spectral libraries are often based on DDA data analyses, e.g., using MaxQuant [9] via a GUI, followed by library generation, e.g., using diapysef [10,11] in a Python shell, and OpenSwath [10] tools on the command line for library refinement. For the analysis of DIA data containing overlapping isolation windows, a demultiplexing step may be required, e.g., during the conversion from vendor-specific file formats to the open mzML [12] format with tools such as msconvert [13]. The identification of peptides in DIA data can be performed using a variety of software suites, e.g., OpenSwathWorkflow [14] followed by false discovery rate (FDR) scoring using PyProphet [15]. The peptide identification and quantification and target decoy scoring, as well as export of the results, can be performed in the standard Windows terminal or an enhanced terminal for Windows such as MobaXterm. The final differential expression analysis can be performed using specialized software such as MSstats [16] in the R programming language. This portrayal serves to illustrate the inherent complexity of modular data analysis in modern DIA proteomics.

The multi-step characteristic of a complete DIA data analysis has encouraged the development of a variety of software options, some of which are particularly powerful in 1 or more of the 3 steps [4,17]. Use of multiple software packages impedes streamlined high-throughput analysis and poses hurdles for software compatibility and reproducibility. Hence, DIA data analysis, especially in the context of powerfully adaptable modular software tools, requires an advanced level of computational skills for software installation, connecting them into analysis workflows and use in the case of command-line–based software. Recent endeavors have used Docker-based structures to distribute pre-assembled and readily usable DIA software bundles, whichill require a high degree of computational, especially programming sk,ills [18–20]. Thus, a hidden requirement for DIA data analysis has been sophisticated bioinformatic skills, owing to the involvement of multiple software tools in a complete analysis and individual analysis steps that are performed using open-source programming languages such as R or Python. To enable straightforward and user-friendly analysis, monolithic software such as Spectronaut and Skyline has been developed [2,21]. However, it remains challenging to embed monolithic software in workflow environments and to enable compatibility and interoperability with other software. Moreover, their design often lacks the ultimate flexibility and tunability of modular software suites such as OpenSwath [10].

OpenSwathWorkflow (OSW) is one of the earliest open-source DIA analysis software suites that supports a large number of functionalities and parameters allowing for a fully customized DIA analysis [22]. Yet, the sophisticated flexibility and numerous parameter options make it difficult to report all crucial settings, e.g., in scientific communications, potentially limiting reproducibility and transparency of DIA analysis. Moreover, OSW by default does not have a GUI, limiting its accessibility and usability to researchers who are familiar with executing software from the command line. The urgent need for a user-friendly and fully customizable DIA analysis pipeline is highlighted by recent endeavors in streamlined DIA analysis options applying OSW [19,20,23].

Here we present a user-friendly repertoire of DIA analysis tools, which can be accessed by a broad user community via the web-based analysis and workflow framework Galaxy [24]. The Galaxy framework makes thousands of bioinformatics tools available to the scientific community without requiring advanced bioinformatics or programming skills. Galaxy analyses are stored in histories, in which all tool names, tool versions, tool parameters, and intermediate data are saved, hence representing an important step for reproducibility. More than 100 public Galaxy servers are available worldwide and offer access to powerful public cloud infrastructure for academic or non-commercial purposes. The European Galaxy instance offers access to 2,775 tools [25] and provides 52 TB of RAM and >12,000 cores (as of November 2021), running on high-performance computing and cloud infrastructures. Into this powerful framework, we have integrated a suite of 11 modular DIA tools based on OpenSWATH [10], diapysef [11], PyProphet [15], and swath2stats [26] (Table 1). Each tool is available as a Conda package and BioContainer and can be installed on any Galaxy instance, enabling deployment for sensitive data use-cases, such as the analysis of clinical samples [27]. Galaxy can be installed as a Docker container; however, a few steps in this workflow require up to hundreds of GB of memory, hence we recommend performing the DIA analysis on the European Galaxy server [24, 28]. Together with existing Galaxy tools, all necessary DIA analysis steps can be executed within Galaxy with high flexibility and in an easily accessible manner. We apply the DIA analysis tools on an *Escherichia coli* K12 and human embryonic kidney 293T spike-in dataset to demonstrate the use of a Galaxy-based DIA analysis pipeline that facilitates standardization and reproducibility and is compatible with the principles of FAIR (findable, accessible, interoperable, and re-usable) data and MIAPE (minimum information about a proteomics experiment) [29,30].

**Table 1:** Overview of newly integrated tools for the DIA data analysis in Galaxy

Integrated tool reference	Function	Galaxy Toolshed
diapysef [10,11]	Spectral library generation	[55]
OpenSwathAssayGenerator [44]	Spectral library refinement	[56]
OpenSwathDecoyGenerator [44]	Spectral library refinement	[57]
TargetedFileConverter [44]	Spectral library conversion	[58]
OpenSwathWorkflow [10, 14]	Peptide identification and quantification in DIA data	[59]
PyProphet merge [15]	Combining individual analysis results to allow for global scoring	[60]
PyProphet subsample [15]	Subsampling of combined analysis results for faster scoring	[61]
PyProphet score [15]	Target-decoy scoring	[62]
PyProphet peptide [15]	Applying computed scores on peptide level	[63]
PyProphet protein [15]	Applying computed scores on protein level	[64]
PyProphet export (includes swath2stats) [15,26]	Export results (Optional: apply swath2stats functionality)	[65]

We integrated 11 tools to enable a complete DIA analysis in Galaxy. Tool names (including the respective references) and their function within the analysis pipeline, as well as a link to the Galaxy toolshed, are provided.

## Methods

### *Escherichia coli* K12 and human embryonic kidney 293T whole-proteome samples

*Escherichia coli*K12 (*E. coli*) and human embryonic kidney 293T (HEK) proteome samples were prepared as previously described [31]. Briefly, cells were lysed using 5% SDS in 50 mM triethylammonium bicarbonate (TEAB) at pH 7.55 by applying sonication (20 cycles with 30/30 sec on/off high energy) with a Bioruptor device (Diagenode, Liège, Belgium). Following centrifugation for 8 min at 13,000*g*, proteins in the supernatant were reduced by incubating with 5 mM TCEP (Sigma-Aldrich, St. Louis, USA) at 95°C for 10 min and subsequently alkylated by incubating with 5 mM IAA (Sigma-Aldrich, St. Louis, USA) at room temperature in the dark. Protein digestion and purification were performed on S-Trap^TM^ micro spin columns (Protifi, Huntington, NY) according to the manufacturer's protocol. After elution, the peptide concentrations were measured using a bicinchoninic acid assay (Thermo Scientific, Rockford, USA) according to the manufacturer's protocol. Different amounts of *E. coli* peptides (0, 0.05, 0.15, 0.4, and 0.8 µg) were added to stable amounts of HEK peptides (2.5 µg), resulting in the following ratios: HEK only, 1:50, 1:17, 1:7, and 1:3. Two replicates of each *E. coli*:HEK ratio were prepared. Samples were vacuum-concentrated until dryness and stored at −80°C until liquid chromatography–tandem mass spectrometry (LC-MS/MS) analysis.

### LC-MS/MS analysis

During mass spectrometry measurements 1 µg of peptides were analyzed on a Q-Exactive Plus mass spectrometer (Thermo Scientific, San Jose, CA) coupled to an EASY-nLC^TM^ 1000 UHPLC system (Thermo Scientific). The analytical column was self-packed with silica beads coated with C18 (Reprosil Pur C18-AQ, d = 3 Å) (Dr. Maisch HPLC GmbH, Ammerbusch, Germany). For peptide separation, a 2-step linear gradient with an increasing amount of buffer B (0.1% formic acid in 80% acetonitrile, Fluka) was applied, ranging from 8% to 43% buffer B over 90 min and from 43% to 65% buffer B in the subsequent 20 min (110 min separating gradient length). Additionally, buffer A and buffer B contained 3% ethylene glycol (final concentration), which has been shown to improve electrospray ionization [32]. For the spectral library 1 representative sample of each *E. coli*:HEK ratio (in total n = 5 samples) was measured using DDA. Briefly, survey scans covering an *m/z* range from 385 to 1,015 *m/z* were performed at 70,000 resolution, an AGC target of 3e6, and a maximum injection time of 50 ms followed by targeting the top 10 precursor ions for fragmentation scans at 17,500 resolution with 1.6 *m/z* isolation windows, a stepped Normalized Collision Energy (NCE) of 25 and 30, and a dynamic exclusion time of 35 s. For all MS2 scans, the intensity threshold was set to 6.3e4, the AGC to 1e5, and the maximum injection time to 160 ms. The 20 *E. coli*:HEK ratios samples were measured using DIA. For DIA 2 cycles of 24 *m/z* broad windows ranging from 400 to 1,000 *m/z* with a 50% shift between the cycles (staggered window schema) were used [33]. MS2 scans were performed at 17,500 resolution, an AGC target of 1e5, and a maximum injection time of 80 ms using a stepped NCE of 25 and 30. After 25 consecutive MS2 scans, an MS1 survey scan was triggered covering the same range and with the same settings as in the DDA measurements.

### Data analysis using Galaxy

The complete data analysis was performed on the European Galaxy server [24]. The analysis history for the spectral library generation and the DIA analysis (including the statistical analysis) have been published via Galaxy [34,35] and can be found in the Additional Files. Briefly, spectral library generation was performed by analyzing 5 DDA measurements representing different *E. coli*:HEK ratios using MaxQuant in Galaxy. A reviewed human protein database containing 20,426 sequences (6 August 2019) and an *E. coli* protein database containing 4,352 sequences (28 March 2019) were retrieved from UniProt. The 5 DDA measurements were specified as fractions to yield a combined peptide and protein identification. For peptide identification, fully tryptic digestion (Trypsin/P) was assumed allowing for ≤1 missed cleavage and ≥1 unique peptide per protein was requested. Carbamidomethylation(C) of cysteine was set as a fixed modification, whereas oxidation(M) on methionine was applied as variable modification. Search results were filtered for 1% FDR on both peptide spectrum match (PSM) as well as protein level. Unique identified peptides, as well as a list of reference peptides (iRT peptides), were used to generate a spectral library with diapysef. The RT alignment method was set to linear. The spectral library was refined using OpenSwathAssayGenerator with the default settings except for a more stringent *m/z* threshold of 0.015 Thompson for both the precursor ion selection as well as the fragment ion annotation. Furthermore, a mass range between 400 and 1,000 Thompson for precursor ions was considered. To allow for subsequent FDR scoring, shuffled decoy transitions were added using the OpenSwathDecoyGenerator and setting the *m/z* threshold to 0.015 Thompson for the fragment ion annotation. In a final step, the spectral library was converted from a tab-separated values (tsv) file format to the peptide query parameter (pqp) format using the TargetedFileConverter. Peptide identification of the DIA measurements was performed using the freshly built spectral library in combination with the same list of reference peptides (iRT peptides) that was already used during the library generation. For the DIA analysis, OpenSwathWorkflow with default settings and a few adjustments was used. The i extraction window was set to 20 ppm on MS/MS and 10 ppm on MS1 level. Within the “Parameters for the RTNormalization for iRT peptides” section the “outlier detection method” was set to “none” and “choose the best peptides based on their peak shape for normalization” was enabled. A minimum number of 7 iRT peptides was requested, and 20 ppm mass tolerance for the iRT transitions was applied. In the “Scoring parameters” section the minimum peak width was set to 5.0 and the computation of a quality value was enabled. The use of mutual information (MI) scores was deactivated. The “Use the retention time normalization peptide MS2 masses to perform a mass correction” was set to “regression_delta_ppm.” OpenSwathWorkflow results of each DIA measurement were combined into a single file using the PyProphet merge tool. Target-decoy scoring of the merged OpenSwathWorkflow results was performed using XGBoost as a classifier in the PyProphet score tool. Computed target-decoy scores were applied on peptide and protein level in an experiment-wide and a global context to estimate protein-level FDR control using the PyProphet peptide and PyProphet protein tool, respectively. DIA analysis results were exported as a tsv file using the PyProphet export tool. Because peptide and protein inference in the global context was conducted, the exported results were filtered to 1% FDR by default. Additionally, the swath2stats functionality was used to provide a summary file and a protein and peptide signal table, as well as an MSstats input tsv file. Measured intensities were normalized using the “equalizeMedians” option in the MSstats tool prior to the differential statistical analysis comparing the different *E. coli*:HEK ratios. Statistical analysis was performed using the MSstats tool as well as a comparison annotation file in 2 different ways: (i) using the MSstats input tsv file generated using the swath2stats functionality and (ii) using the PyProphet export tsv file and an MSstats sample annotation file. For the 2 approaches, the “input source” parameter needs to be adjusted and set to “MSstats 10 column format” when using the swath2stats prepared tsv file or “OpenSWATH” when using the PyProphet tsv file. Coefficients of variation (CV) of *E. coli* proteins in the spike-in samples and of human proteins in the HEK-only samples were computed on the basis of the normalized protein quantifications using R (v 4.1.2) in RStudio (Version 1.1.463) and visualized with ggplot2 (v 3.3.5) [36].

## Findings

### Galaxy enables easily accessible, straightforward, and reproducible DIA data analysis

**Figure 1:**
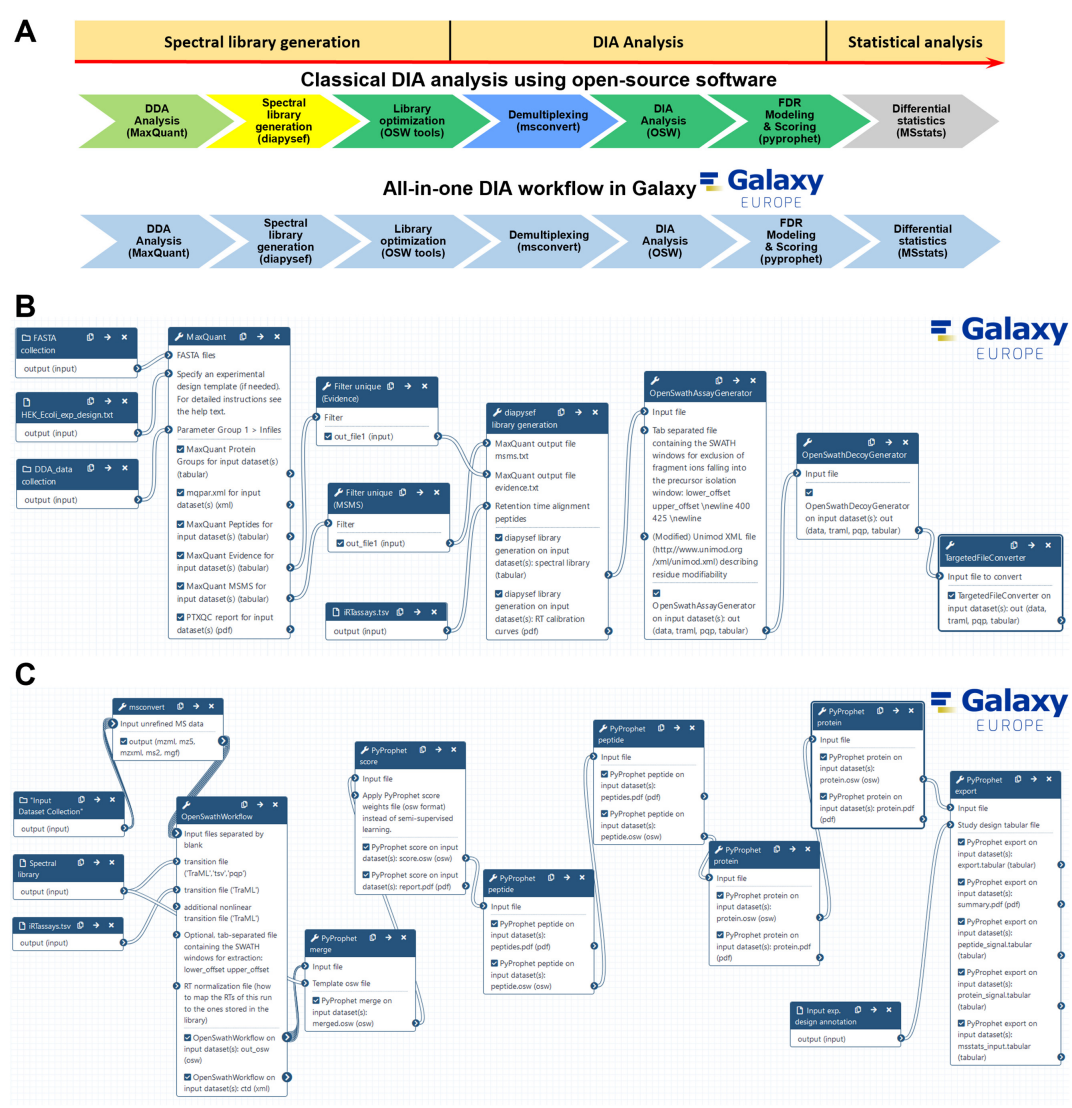
Introducing an all-in-one DIA analysis solution by implementing all necessary tools for a DIA analysis into the Galaxy framework. (A) Schematic overview of a classic data-independent acquisition (DIA) analysis workflow as compared to the here-introduced all-in-one workflow in the Galaxy framework. The classic DIA workflow includes different software environments and operating system requirements as indicated by a different color (light green: local MaxQuant analysis; yellow: diapysef Python shell; dark green: MobaXterm enhanced terminal for Windows; blue: local msconvert; grey: MSstats in Rstudio), whereas all necessary tools are now implemented into the Galaxy framework. A complete DIA analysis can be divided into 3 steps: (i) spectral library generation, (ii) peptide and protein identification and quantification in DIA data, and (iii) statistical analysis to identify differentially expressed proteins. (B) Generation of a spectral library based on the analysis of data-dependent acquisition (DDA) analysis shown as Galaxy workflow. (C) DIA data analysis shown as Galaxy workflow.

Here we present an all-in-one DIA analysis pipeline within the Galaxy framework, enabling easy access to a suite of advanced software tools and providing sufficient computational resources for large-scale DIA data analyses. We developed and implemented all necessary tools required for a complete DIA data analysis into the Galaxy framework (Fig. 1A and Table 1). The newly implemented tools were integrated with state-of-the-art proteomic Galaxy tools such as MaxQuant [9], MSstats [16], and basic text manipulation tools to build a functional DIA analysis pipeline. All newly integrated DIA tools are based on open-source software such as diapysef [11], OpenSwath [10], PyProphet [15], and swath2stats [26]. The tools were built in a modular way, allowing a fully customized analysis. Each analysis step can be executed individually or assembled as a workflow in the Galaxy platform, facilitating a streamlined and straightforward analysis (Fig. 1A) [14,22,37]. All parameter options of the original software can be modified via the Galaxy GUI, providing a maximum of user-adjustable configurations for fine-tuning of analysis. We consider archiving of such details to be relevant for reproducibility; deposition and sharing of entire Galaxy workflows is a straightforward and integrated way of doing so. Published Galaxy histories include complete provenance data, allowing users to reproduce the same analysis that has been published. Thus, version control and version archiving constitute a major feature of this approach.

We present a Galaxy-based, complete DIA analysis pipeline that consists of 3 major parts: (i) spectral library generation using DDA data (Fig. 1B) [38], (ii) peptide (and protein) identification and quantification in DIA data using the aforementioned spectral library (Fig. 1C) [39], and (iii) statistical analysis (Supplementary Fig. 1) [40,41]. The workflows for each step have been published via Galaxy and can be downloaded and adjusted or directly run in Galaxy [38–41].

We wish to emphasize that embedding these tools in Galaxy not only enables user-friendly use but also fosters a new level of reporting and reproducibility in DIA proteomics: the tools as such may be combined in different ways and many tools provide an array of user-adjustable fine-tuning parameters. For illustration, the basic DIA analysis workflow (provided as training and discussed in further detail below) includes, among others, the 3 tools OpenSwathWorkflow [14], PyProphet [15] score, and PyProphet [15] peptide, each of which have >10 fine-tuning settings. This results in numerous possible parameter combinations, which, to our knowledge, are rarely reported in detail. The Galaxy framework addresses this issue by making it possible to deposit and share entire workflows (including complete provenance data), also in the context of scientific publications. We consider this feature to be a major benefit of our approach of integrating a DIA processing suite in Galaxy.

### Democratizing data-independent acquisition analysis via the Galaxy framework

The web-based access and GUI in Galaxy empowers a broad community of researchers to perform DIA data analysis. All DIA Galaxy tools are pre-installed and ready to be used on several public Galaxy servers, e.g., the European Galaxy server [24]. Running on public clouds, these Galaxy servers provide an immense computational power that enables many DIA analyses to be run in parallel without the need to invest or block private computing resources. Most of the software that we integrated into Galaxy is normally only usable with basic programming skills in R or Python and thus excludes many proteomics researchers from using them. With the integration into Galaxy, these software tools are now usable by a much broader community via Galaxy's GUI, which allows all input files and parameters to be specified and analysis workflows to be built on the basis of modular tools.

In the following sections, we present more detailed insight into the various steps of Galaxy-supported DIA analysis using the tool suite.

### Spectral library generation–based on data-dependent acquisition measurements

**Figure 2:**
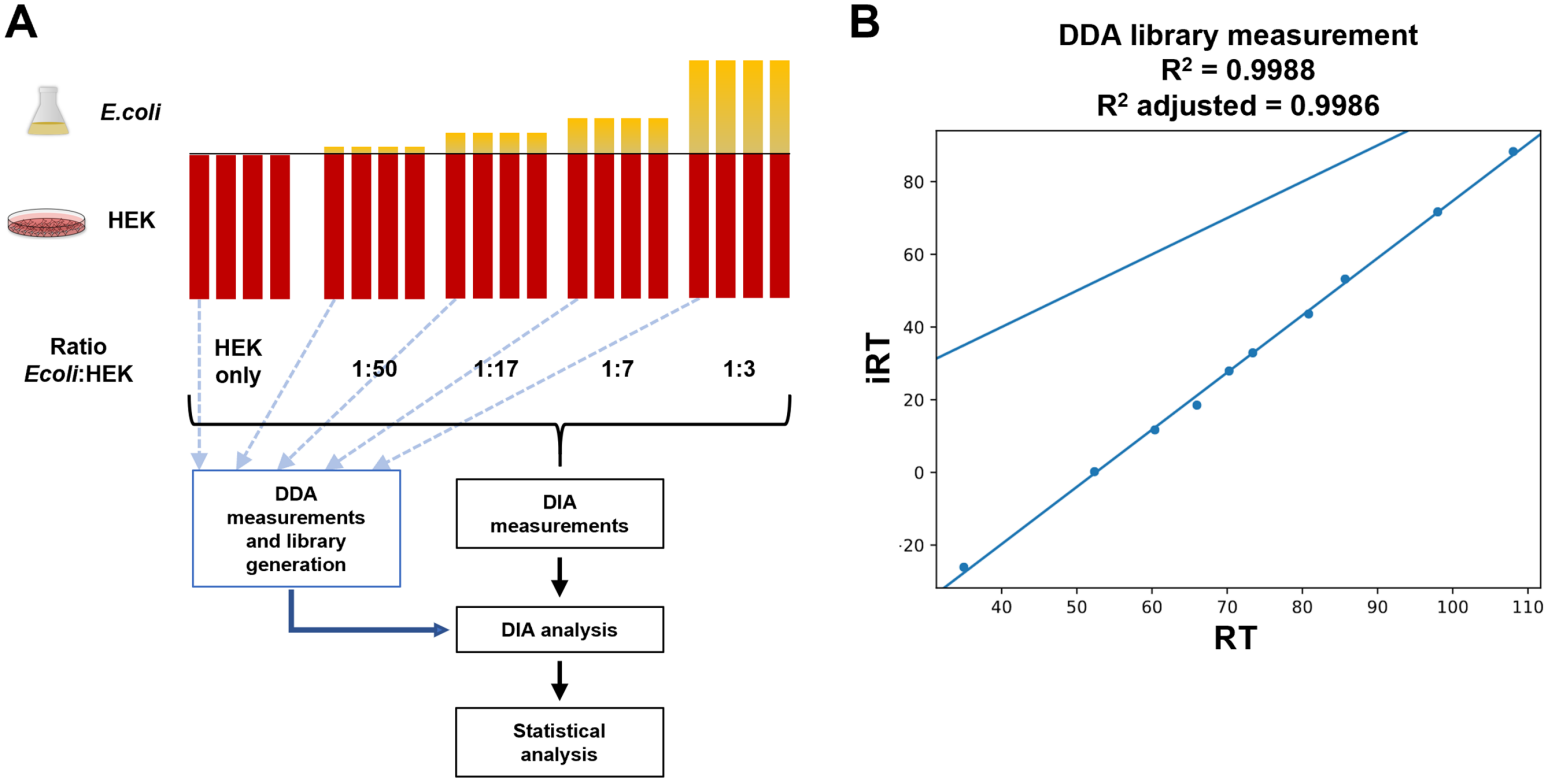
Analysis of a DIA spike-in dataset in Galaxy. (A) Experimental design of a spike-in dataset based on equal amounts of HEK proteome and known spike-in amounts of *E. coli* proteome. For spectral library generation, 1 representative sample of each mixture was measured using data-dependent acquisition (DDA). Each *E. coli*:HEK ratio was measured in 4 replicates using data-independent acquisition (DIA). DIA analysis was performed on the basis of the spectral library and the individual DIA measurements followed by statistical analysis to identify differentially expressed proteins. (B) Retention time (RT) alignment plot of the measured RT and respective indexed retention time (iRT) of reference peptides (iRT peptides) during the generation of the spectral library (exemplarily shown for 1 of the DDA measurements). All measured RTs are converted to iRTs on the basis of the linear regression of the reference peptides (*R*² and *R*² adjusted for the linear regression are shown above the plot).

A spectral library is generated with the newly integrated diapysef [11] tool using either the proteotypic peptides or the unfiltered MaxQuant [9] results in combination with a list of reference peptides, to which the RTs of all identified peptides will be aligned using either a linear or a non-linear regression [42,43]. The diapysef tool in Galaxy automatically generates a pdf file containing the RT calibration curves, highlighting the identified reference peptides and the respective linear or non-linear regression fit (Fig. 2B). The calibration curves provide a valuable overview of the suitability of the reference peptide with regards to their linear elution, as well as the reproducibility of the identification and elution in the analyzed samples/fractions. To improve the sensitivity and selectivity for the detection of typical peptides the spectral library can be refined using the OpenSwathAssayGenerator tool [44]. Briefly, the number of transitions per precursor ion is reduced and precursors can be filtered to fit the covered mass-to-charge range of the DIA measurements (typically between 400 and 1,000 *m/z*). To enable FDR-based scoring, an equal number of decoy transitions can be added to the spectral library using the OpenSwathDecoyGenerator tool. In an optional step, the spectral library can be converted to the required format (traml, tsv, or pqp) using the TargetedFileConverter tool. In particular, for the generation of result files in the osw format using OpenSwathWorkflow, the spectral library is required as a pqp file.

### OpenSwath in Galaxy allows for the versatile, reproducible, and robust DIA analysis of large proteomic cohorts [10]

**Figure 3:**
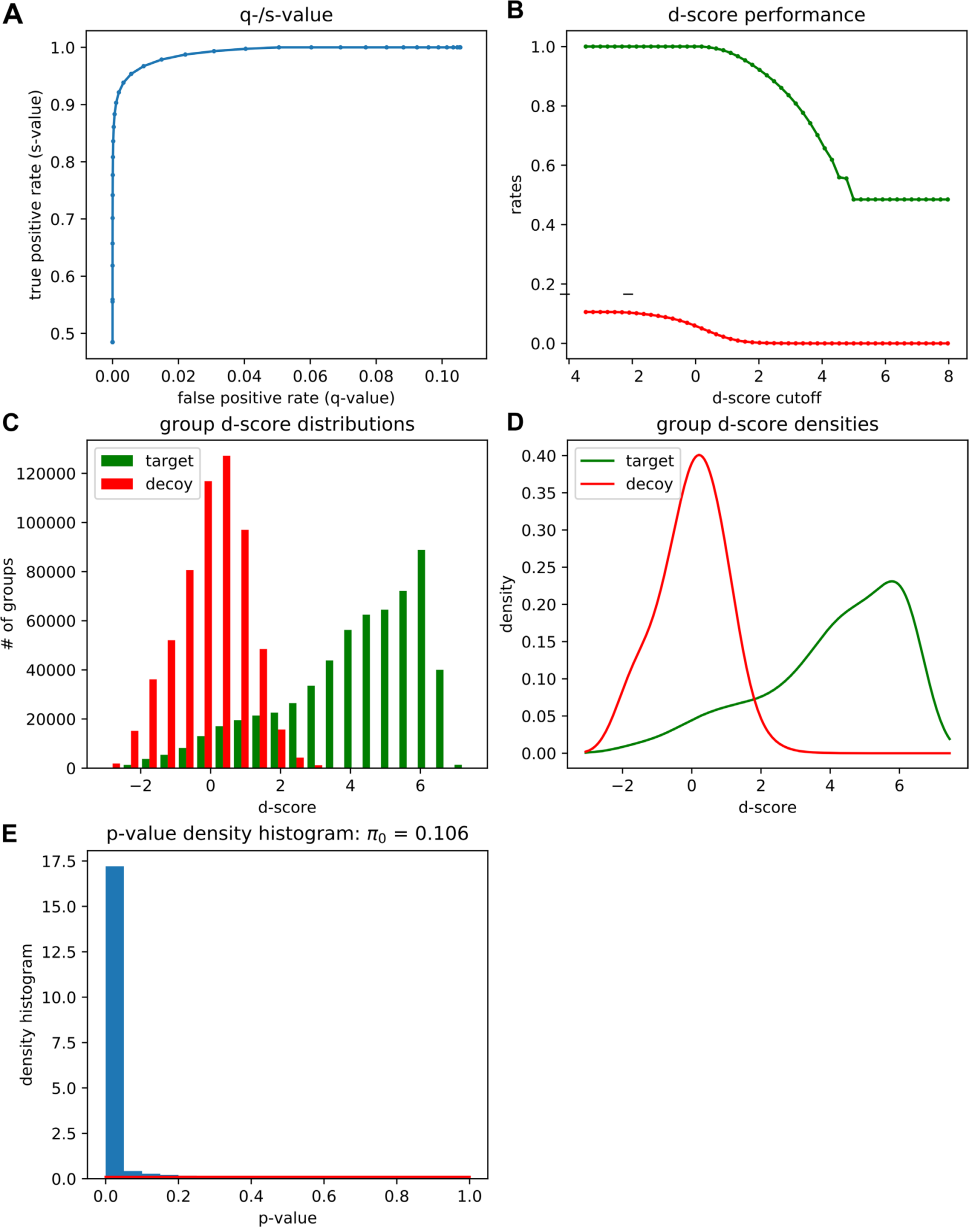
Overview of target-decoy scoring using PyProphet score during the DIA analysis in Galaxy. (A) Receiver operating characteristic (ROC) curve highlighting the sensitivity and specificity of the target-decoy scoring. (B) Plot showing the discriminatory score (d-score) performance between the target (green) and decoy (red) precursors. (C) Bar plot and (D) density plot showing d-score distribution among target and decoy precursors. (E) Histogram showing the distribution of *P*-values computed on the basis of the target-decoy scoring.

The peptide identification and quantification of the individual DIA measurements are performed in the OpenSwathWorkflow [14] (OSW) tool in Galaxy using the freshly built spectral library and a list of reference peptides (RT peptides), as well as the demultiplexed DIA files in the open mzML [12] format. In most studies, multiple DIA measurements are performed and the different samples should be compared qualitatively and/or quantitatively. Therefore, the individual OSW result files can be merged using the spectral library as a template in the PyProphet [15] merge tool. The target-decoy scoring is performed by applying semi-supervised learning and an error rate estimation using the PyProphet [15] score tool on the merged OSW results. Notably, the semi-supervised learning and error rate estimation of a merged file containing several hundreds of individual DIA measurements can require considerable computational resources. On the European Galaxy instance, the PyProphet score tool currently uses up to 400 GB of RAM, which can be further expanded up to 1 TB if required [45]. To decrease the analysis time of the semi-supervised learning, the merged OSW results can be first subsampled using the PyProphet [15] subsample tool and subsequently scored using the PyProphet score tool [46]. The computed scores can be applied to the complete merged OSW results. The PyProphet score tool in Galaxy generates an overview of the sensitivity and specificity of the target-decoy scoring, as well as a visualization of the distributions of target and decoy transitions (Fig. 3). To conduct peptide and protein inference in run-specific, experiment-wide, or global context the tools PyProphet [15] peptide and PyProphet [15] protein can be used, respectively [46]. Each step will generate an overview of the scores and the resulting target-decoy distributions (Supplementary Figs 2–5). Afterward, peptide identification and quantification results can be exported as a tsv file using the PyProphet [15] export tool in Galaxy. Furthermore, we integrated the swath2stats [26] functionality into the PyProphet export tool, allowing the user to visualize the analysis results and to further process the results, e.g., providing an MSstats [16] compatible input tsv.

### MSstats enables statistical relative quantification of proteins and peptides in DIA proteomics

In most quantitative proteomic studies, a statistical analysis is performed to identify significantly altered peptide and protein profiles between different samples. MSstats [16] is a specialized R programming language package for the statistical analysis of proteomic data that has recently been implemented as a Galaxy tool. Differential expression analysis of DIA data can be performed using the swath2stats [26] processed tsv file or by using the non-processed output of the PyProphet [15] export tool in combination with a sample annotation and a comparison matrix file with the MSstats tool. The comparison matrix contains information about the conditions that should be compared against each other during differential statistical analysis.

## Accessibility and Training

Our Galaxy DIA tools are accompanied by hands-on training material, which we have developed and made available via the central Galaxy Training Network [47,48]. The DIA analysis training material is split into 3 major steps: (i) generation of a spectral library [49], (ii) DIA data analysis [50], and (iii) statistical analysis [51]. The web addresses of the online training are provided as references [49–51]. Each training contains step-by-step information about the data handling and processing, as well as a brief introduction to the theory and principles of the respective analysis step. A concise set of training data is provided via a publicly available deposit [31,52]. Users can directly load the training data into a Galaxy history via the Galaxy URL upload functionality. Using either the training data or their own input data, users can follow the step-by-step introduction provided in the training material. To increase the learning experience through active participation each training includes questions regarding intermediate results based on the provided training data. Of note, intermediate results of rather time-consuming analysis steps are provided, limiting the required execution time of each training. With the extensive set of Galaxy training material for a complete DIA analysis, we wish to enable efficient online self-education of researchers around the world, a topic that has gained increasing interest due to an avalanche of recent, pandemic-related travel restrictions [53].

## Case Study

**Figure 4:**
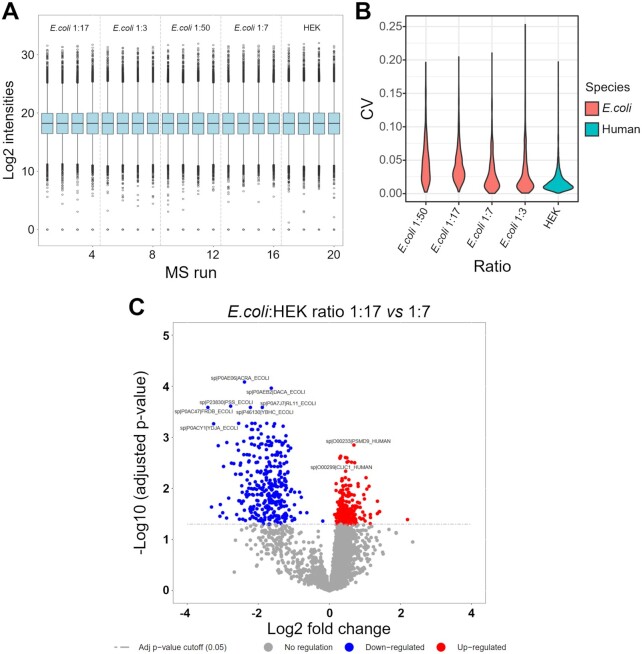
Protein quantification results obtained using the DIA analysis tools in Galaxy. (A) Box plot showing the distribution and median value of global median normalized log_2_ protein intensities. (B) Violin plot illustrating the distribution of coefficients of variation (CV) of the log_2_-transformed protein intensities for each condition (here *E. coli*:HEK ratios) across replicates (each n = 4). For *E. coli*–containing mixture only *E. coli* proteins were used (red), and for the HEK replicates only human proteins were used (blue). (C) Volcano plot showing −log_10_ adjusted *P*-values against log_2_ fold changes, highlighting differentially expressed proteins comparing the 2 *E. coli*:HEK ratios 1:17 vs 1:7. Upregulated proteins are shown in red whereas downregulated proteins are shown in blue. The significance threshold is indicated as dashed line at a adjusted P-value of 0.05.

To illustrate the functionality and utility of our DIA analysis pipeline, we analyzed a DIA dataset, representing a human cell line proteome (HEK cells) with known spike-in amounts of a distinguishable bacterial *E. coli* proteome (Fig. 2A). Additionally, all samples contain a set of 11 synthetic reference peptides for the RT alignment [42]. The dataset includes *E. coli*:HEK ratios ranging from 1:50 to 1:3, reflecting a dynamic range of the altered protein abundances. For each *E. coli*:HEK ratio n = 4 replicates were measured, resulting in a total of 20 DIA measurements. For spectral library generation, 1 representative sample of each *E. coli*:HEK ratio was measured using DDA and analyzed with the MaxQuant tool in Galaxy. The Galaxy framework allows all compatible tools to be combined. We applied a basic Galaxy text manipulation tool to filter the peptide and protein identifications tsv file for proteotypic peptides to avoid ambiguous peptides that potentially originate from various proteins. The spiked-in reference peptides elute linearly as highlighted by the RT calibration curves (Fig. 2B, exemplarily shown for 1 of the DDA measurements). The staggered window schema of the DIA measurements required demultiplexing before the analysis with OpenSwathWorkflow [14], which was performed using the msconvert [13] tool in Galaxy. The analysis of the 20 DIA measurements with the OpenSwathWorkflow tool in Galaxy results in the sensitive and selective identification of target transitions (Fig. 3A). Furthermore, target and decoy transitions show distinct distributions based on the computed d-scores with the PyProphet [15] score tool (Fig. 3B–D). We identified and quantified 25,000–27,000 peptides derived from 4,800–5,000 proteins in each of the individual *E. coli*:HEK samples (Table 2). Prior to the statistical analysis measured intensities were normalized using the “equalizeMedians” option in the MSstats tool (Fig. 4A). As expected, the CV distribution for *E. coli* proteins slightly increases with reduced *E. coli* spike-in amounts (Fig. 4B). Human proteins detected in the HEK-only samples show the lowest CV distribution. Differential expression analysis using the MSstats [16] tool revealed significantly dysregulated proteins between the different *E. coli*:HEK ratios. As expected when comparing the 1:17 against the 1:7 *E. coli*:HEK ratio, multiple *E. coli* proteins are significantly downregulated in the 1:17 *E. coli*:HEK samples (Fig. 4C). Furthermore, some human proteins seem to be upregulated in this comparison, which might be due to displacement effects by the added amount of *E. coli* proteome (Supplementary Fig. S6). While having constant amounts of HEK proteome as a background an increasing spike-in amount of *E. coli* proteins might result in fewer detected human proteins, as well as displacement effects during the chromatography and ion suppression during the ionization. Even when comparing the 2 lowest *E. coli*:HEK ratios (1:50 vs 1:17) significantly dysregulated *E. coli* proteins can be detected, highlighting the overall functionality as well as a suitable sensitivity of the DIA analysis pipeline in Galaxy (Supplementary Fig. S7). The complete analysis was performed on the European Galaxy instance [24]. All analysis histories, as well as workflows, are available in the supporting information of this publication to ensure full reproducibility of the presented results. Accurate, transparent, and complete sharing of parameters and whole analysis is simplified by Galaxy's intrinsic features for sharing and publishing. Shared histories and workflows, as well as the Galaxy software, fulfill the FAIR principles [29].

**Table 2:** Overview of identified and quantified precursors, peptides, and proteins

*E. coli*:HEK ratio	Replicate	Precursors	Peptides	Proteins
1:17	1	28,792	27,117	4,979
	2	28,697	27,040	4,972
	3	28,673	27,024	4,970
	4	28,641	27,003	4,989
1:3	1	28,711	27,060	5,011
	2	28,690	27,043	5,000
	3	28,672	27,028	5,006
	4	28,669	27,035	4,996
1:50	1	28,191	26,576	4,906
	2	28,255	26,636	4,914
	3	28,231	26,595	4,919
	4	28,192	26,577	4,916
1:7	1	28,833	27,160	4,994
	2	28,791	27,123	5,006
	3	28,804	27,136	5,004
	4	28,837	27,166	5,011
HEK only	1	27,166	25,669	4,843
	2	27,182	25,683	4,862
	3	27,176	25,663	4,869
	4	27,099	25,600	4,858

DIA analysis results were filtered at 1% FDR on peptide and protein level during export using the PyProphet export tool. In combination with a sample annotation file, the swath2stats functionality was applied, yielding an overview of identified and quantified precursors, peptides, and proteins in each sample.

## Conclusions

To conclude, our DIA Galaxy tools and workflows represent a powerful and user-friendly software solution for the analysis of large-scale DIA experiments. We implemented DIA analysis tools based on open-source software such as OpenSwath, PyProphet, diapysef, and swath2stats that can be integrated with existing tools, providing a flexible and modular analysis pipeline. Moreover, the tools can be assembled into complete DIA analysis workflows, promoting straightforward and reproducible analysis of large sample cohorts. All tools are accessible via the Galaxy system of GUIs and have access to public clouds. The web-based access in Galaxy and the extensive training material empower a broad community of researchers to perform their DIA analysis, without the need for enhanced computational skills and resources. Complete analysis histories and workflows can be shared and published via Galaxy, promoting transparent and reproducible DIA analysis. By integrating a suite of modular DIA tools in Galaxy and presenting fit-for-purpose, readily usable DIA workflows, we make the abilities and reproducibility of the Galaxy infrastructure accessible to the DIA proteomics community.

## Availability of Supporting Source Code and Requirements

- Project name: Data independent acquisition proteomics workbench
- Project homepage: https://github.com/galaxyproteomics/tools-galaxyp
- Galaxy Toolshed: https://toolshed.g2.bx.psu.edu/
- Operating system: Linux
- Training repository: https://training.galaxyproject.org/training-material/topics/proteomics/
- RRID:SCR_021862
- License: MIT

## Data Availability

The Galaxy workflows underlying this article are available at the Galaxy Europe website, as follows: workflow to generate a spectral library at https://usegalaxy.eu/u/matthiasfahrner/w/dia-lib-hek-ecoli-3eg-data; to perform DIA analysis at https://usegalaxy.eu/u/matthiasfahrner/w/dia-analysis-using-hek-ecoli-3-eg-data; to perform the statistical analysis (i) using swath2stats converted MSstats input at https://usegalaxy.eu/u/matthiasfahrner/w/hek-ecoli-dia-statistics-swath2stats-3eg-data and (ii) using pyprophet export tsv at https://usegalaxy.eu/u/matthiasfahrner/w/hek-ecoli-dia-statistics-3eg-data-1. The Galaxy history of the spectral library generation is available at https://usegalaxy.eu/u/matthiasfahrner/h/dia-lib-hek-ecoli-3eg-data, and the Galaxy history of the DIA analysis including statistical analysis at https://usegalaxy.eu/u/matthiasfahrner/h/dia-analysis-statistics-hek-ecoli-3eg-data. Mass spectrometry data have been deposited and are available in the MassIVE repository with accession MSV000087859. https://massive.ucsd.edu/ProteoSAFe/dataset.jsp?task=0aa8d075dc2e4abf98832db002f03ea6. Other data further supporting this work are openly available in the *GigaScience* repository, GigaDB [54].

## Additional Files

**Supplementary Figure S1**.Galaxy workflows for statistical analysis of DIA data. Galaxy workflow for statistical analysis of DIA data with MSstats using a group comparison matrix file and (A) the swath2stats-processed PyProphet export results or (B) the direct PyProphet export results and a sample annotation file.

**Supplementary Figure S2**.Overview of target-decoy scoring results using PyProphet peptide with experiment-wide peptide-level error rate control. (A) Receiver operating characteristic (ROC) curve highlighting the sensitivity and specificity of the target-decoy scoring. (B) Plot showing the discriminatory score (d-score) performance between the target (green) and decoy (red) peptides. (C) Bar plot and (D) density plot showing d-score distribution among target (green) and decoy (red) peptides. (E) Histogram showing the distribution of *P*-values computed on the basis of the target-decoy scoring.

**Supplementary Figure S3**.Overview of target-decoy scoring results using PyProphet peptide with global peptide-level error rate control. (A) Receiver operating characteristic (ROC) curve highlighting the sensitivity and specificity of the target-decoy scoring. (B) Plot showing the discriminatory score (d-score) performance between the target (green) and decoy (red) peptides. (C) Bar plot and (D) density plot showing d-score distribution among target (green) and decoy (red) peptides. (E) Histogram showing the distribution of *P*-values computed on the basis of the target-decoy scoring.

**Supplementary Figure S4**.Overview of target-decoy scoring results using PyProphet protein with experiment-wide protein-level error rate control. (A) Receiver operating characteristic (ROC) curve highlighting the sensitivity and specificity of the target-decoy scoring. (B) Plot showing the discriminatory score (d-score) performance between the target (green) and decoy (red) proteins. (C) Bar plot and (D) density plot showing d-score distribution among target (green) and decoy (red) proteins. (E) Histogram showing the distribution of *P*-values computed on the basis of the target-decoy scoring.

**Supplementary Figure S5**.Overview of target-decoy scoring results using PyProphet protein with global protein-level error rate control. (A) Receiver operating characteristic (ROC) curve highlighting the sensitivity and specificity of the target-decoy scoring. (B) Plot showing the discriminatory score (d-score) performance between the target (green) and decoy (red) proteins. (C) Bar plot and (D) density plot showing d-score distribution among target (green) and decoy (red) proteins. (E) Histogram showing the distribution of *P*-values computed on the basis of the target-decoy scoring.

**Supplementary Figure S6**.Differential statistical analysis results comparing the *E. coli*:HEK ratios 1:17 against 1:7. Volcano plot showing −log_10_ adjusted p-values against log_2_ fold changes, highlighting differentially expressed proteins comparing the 2 *E. coli*:HEK ratios 1:17 vs 1:7. Significantly dysregulated proteins are colored by species (human, red and *E. coli*, blue).

**Supplementary Figure S7**.Differential statistical analysis results comparing the *E. coli*:HEK ratios 1:50 against 1:17. Volcano plot showing −log_10_ adjusted *P*-values against log_2_ fold changes, highlighting differentially expressed proteins comparing the 2 *E. coli*:HEK ratios 1:50 vs 1:17.

giac005_GIGA-D-21-00223_Original_Submission

giac005_GIGA-D-21-00223_Revision_1

giac005_GIGA-D-21-00223_Revision_2

giac005_Response_to_Reviewer_Comments_Revision_1

giac005_Reviewer_1_Report_Original_Submission Paul Stewart -- 8/13/2021 Reviewed

giac005_Reviewer_2_Report_Original_Submission Peter Horvatovich -- 9/5/2021 Reviewed

giac005_Reviewer_2_Report_Revision_1 Peter Horvatovich -- 12/5/2021 Reviewed

giac005_Supplemental_Files

## Abbreviations

AGC: automatic gain control; CV: coefficient of variation; DDA: data-dependent acquisition; DIA: data-independent acquisition; FAIR: findable, accessible, interoperable; and re-usable; FDR: false discovery rate; GUI: graphical user interface; IAA: Iodoacetamide; LC-MS/MS: liquid chromatography–tandem mass spectrometry; MIAPE: minimum information about a proteomics experiment; OSW: OpenSwathWorkflow; PQP: peptide query parameter; RAM: random access memory; RT: retention time; SDS: sodium dodecyl sulfate; TCEP: Tris(2-carboxyethyl)phosphin, TSV: tab-separated values.

## Competing Interests

The authors declare that they have no competing interests.

## Funding

O.S. acknowledges funding by the Deutsche Forschungsgemeinschaft (DFG, SCHI 871/17–1, SCHI 871/15–1, GR 4553/5–1, PA 2807/3–1, NY 90/6–1, INST 39/1244–1 (P12), INST 39/766–3 (Z1), 423813989/GRK2606 “ProtPath”; Project-ID 441 891 347-SFB-1479; Project-ID 431 984 000–SFB 1453), the ERA PerMed program (BMBF, 01KU1916, 01KU1915A), the German-Israel Foundation (grant No. 1444), the German Consortium for Translational Cancer Research (project Impro-Rec), and the Fördergesellschaft Forschung Tumorbiologie (projects ILBIG and NACT).

## Authors' Contributions

M.F. conceived the project, tested the tools, and developed the training material and the case study. M.C.F. developed the Galaxy tool wrappers and the training material and contributed to the conceptualization. B.A.G. and M.B. developed the Galaxy tool wrapper. H.R. developed parts of the applied software. B.G. and O.S. contributed to the conceptualization, methodology, and funding acquisition. M.F. wrote the manuscript. All authors critically read and approved the manuscript's contents.
