# Supplementary figures and images for: Democratizing data-independent acquisition proteomics analysis on public cloud infrastructures via the Galaxy framework
Source: Gigascience. 2022 Feb 15;11:giac005. doi: 10.1093/gigascience/giac005 (PMC8848309; doi:10.1093/gigascience/giac005)

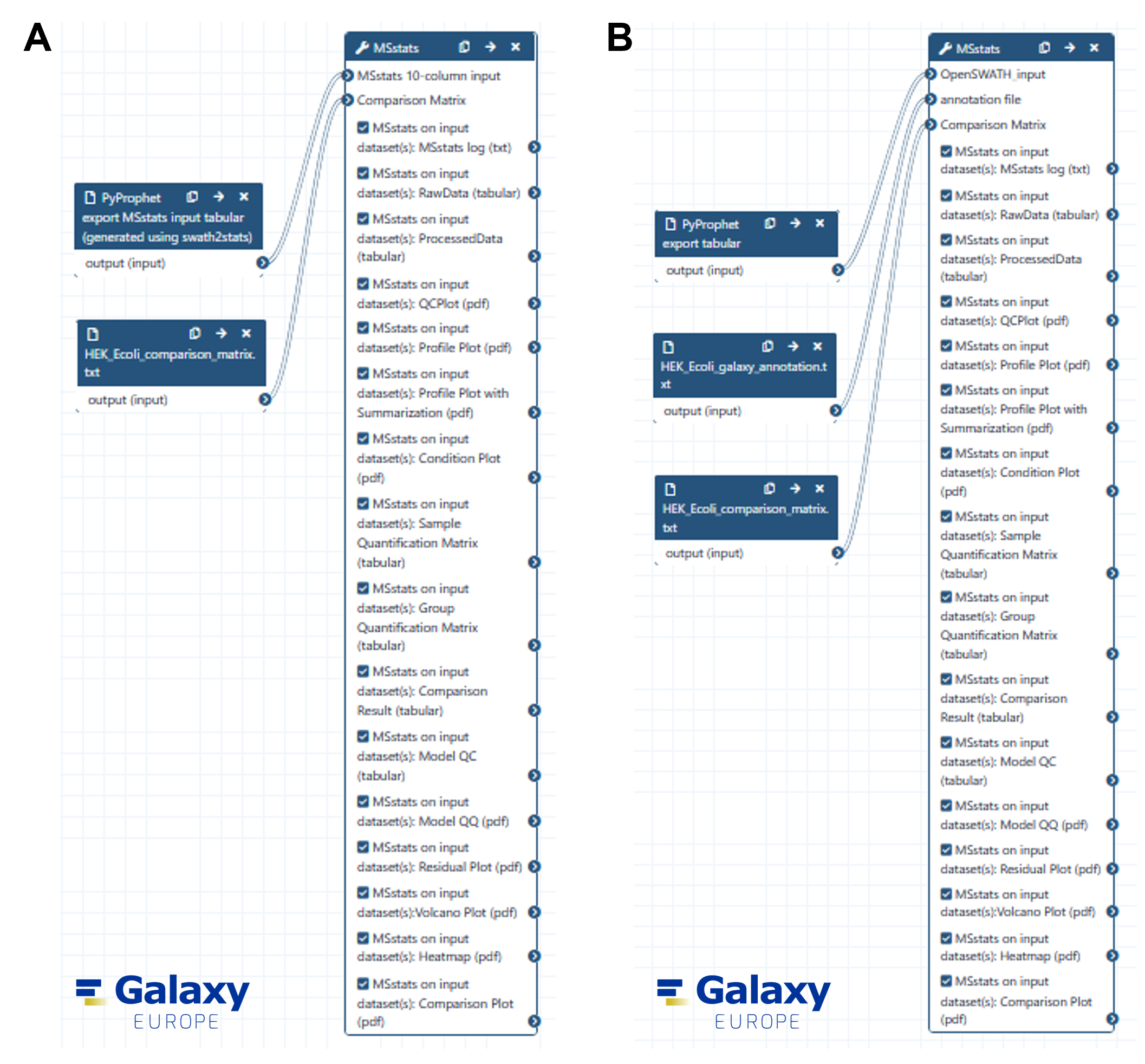

Supplement: giac005_Supplemental_Files [file giac005_supplemental_files.zip › Figure1_Supplementary.png]

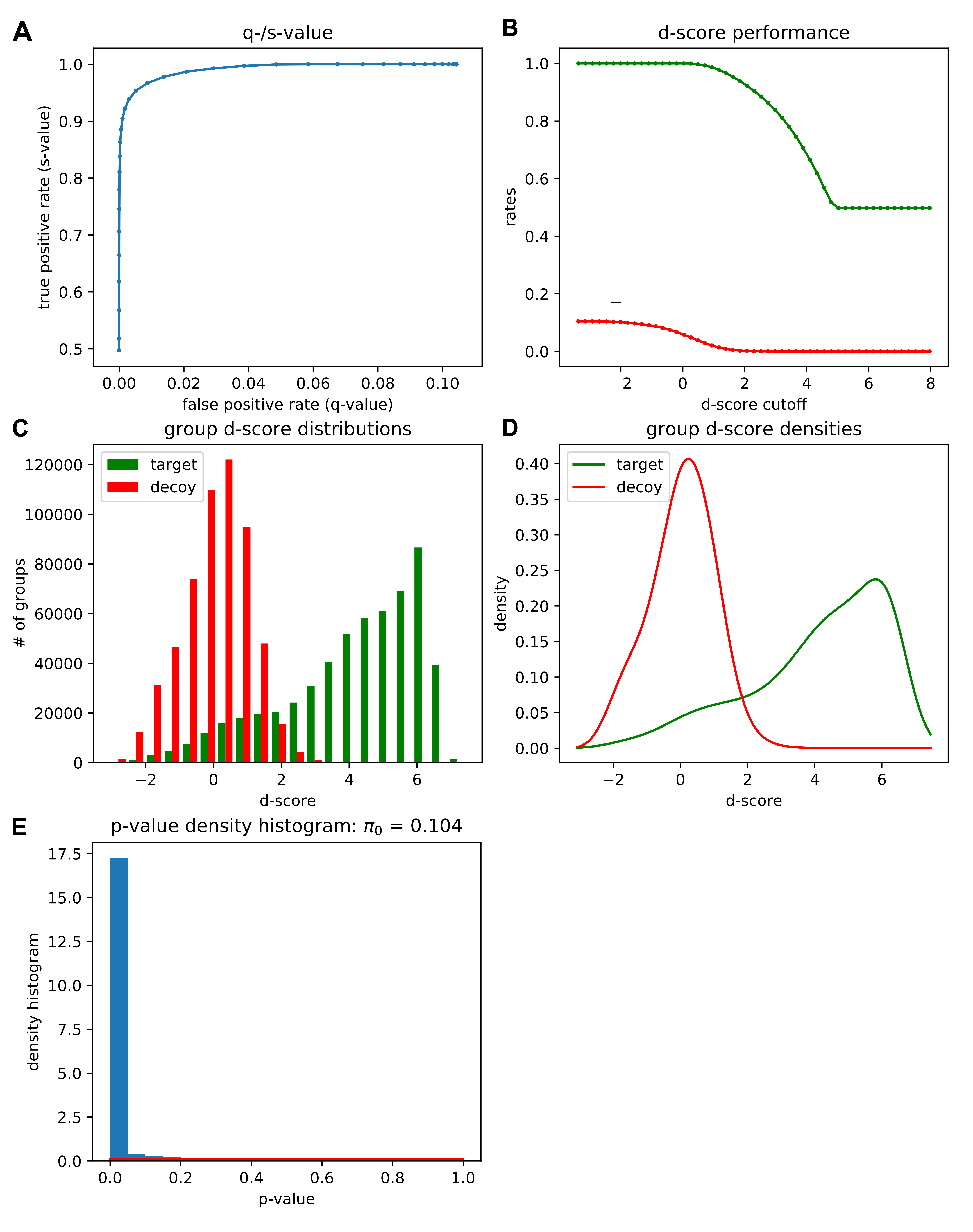

Supplement: giac005_Supplemental_Files [file giac005_supplemental_files.zip › Figure2_Supplementary.png]

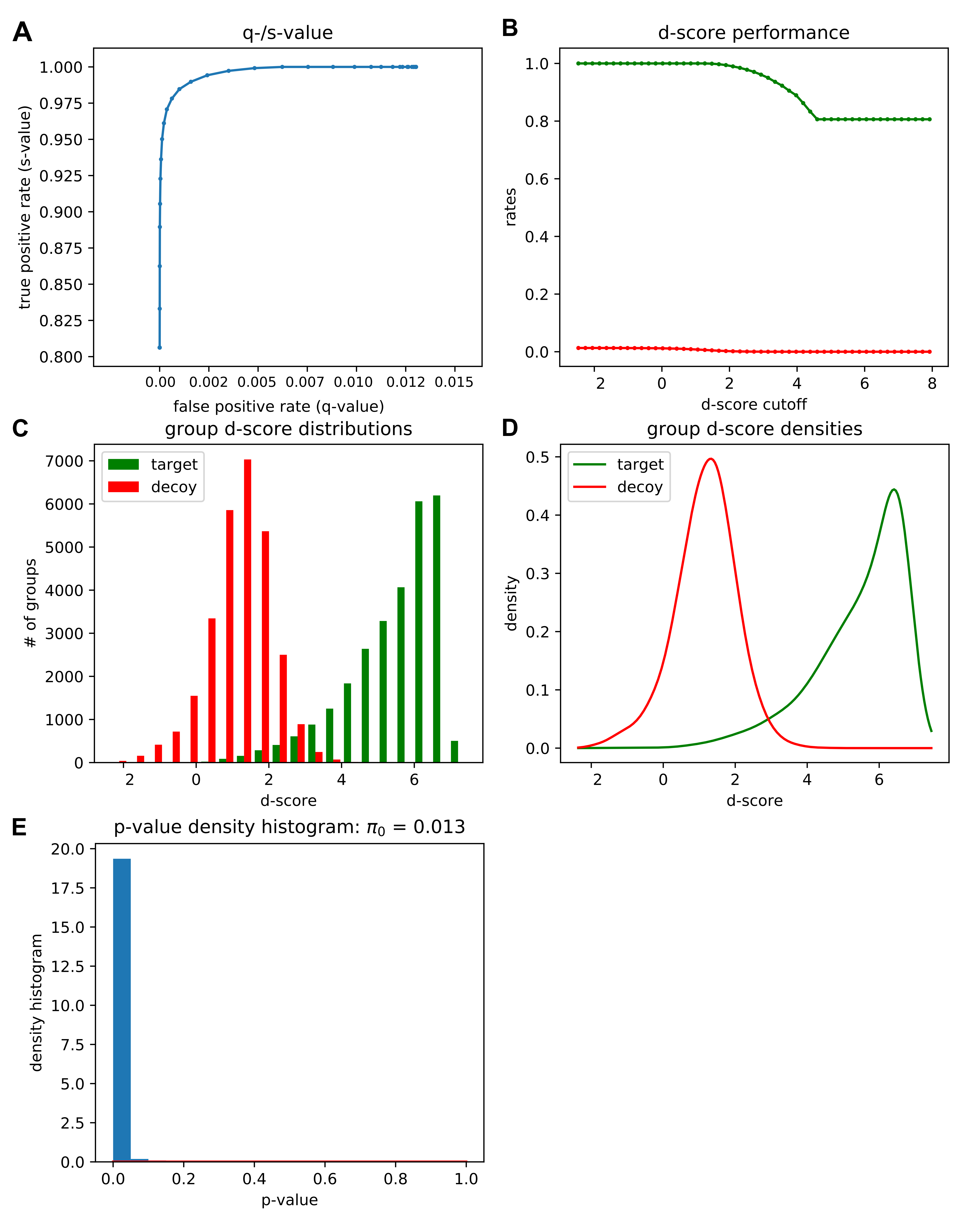

Supplement: giac005_Supplemental_Files [file giac005_supplemental_files.zip › Figure3_Supplementary.png]

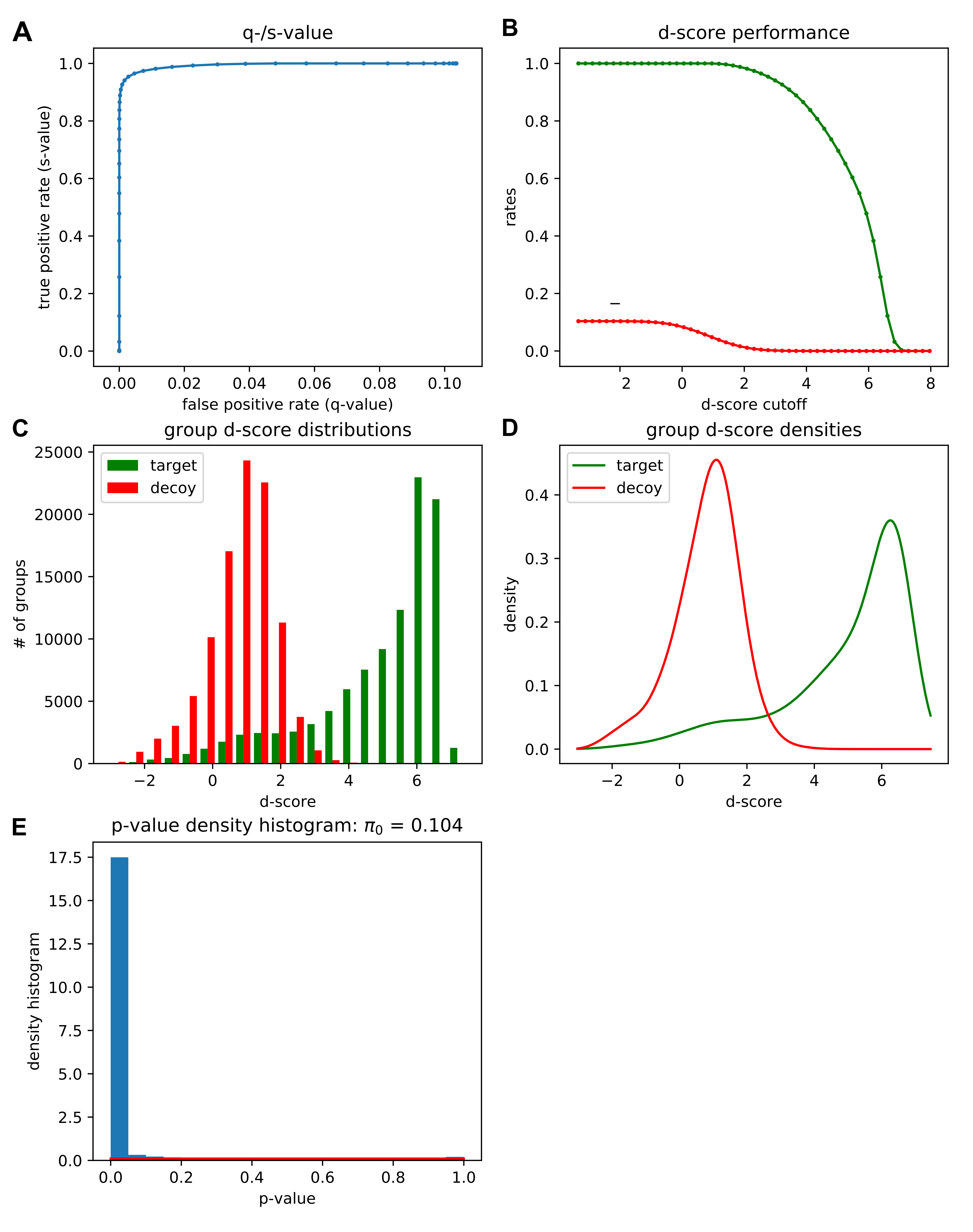

Supplement: giac005_Supplemental_Files [file giac005_supplemental_files.zip › Figure4_Supplementary.png]

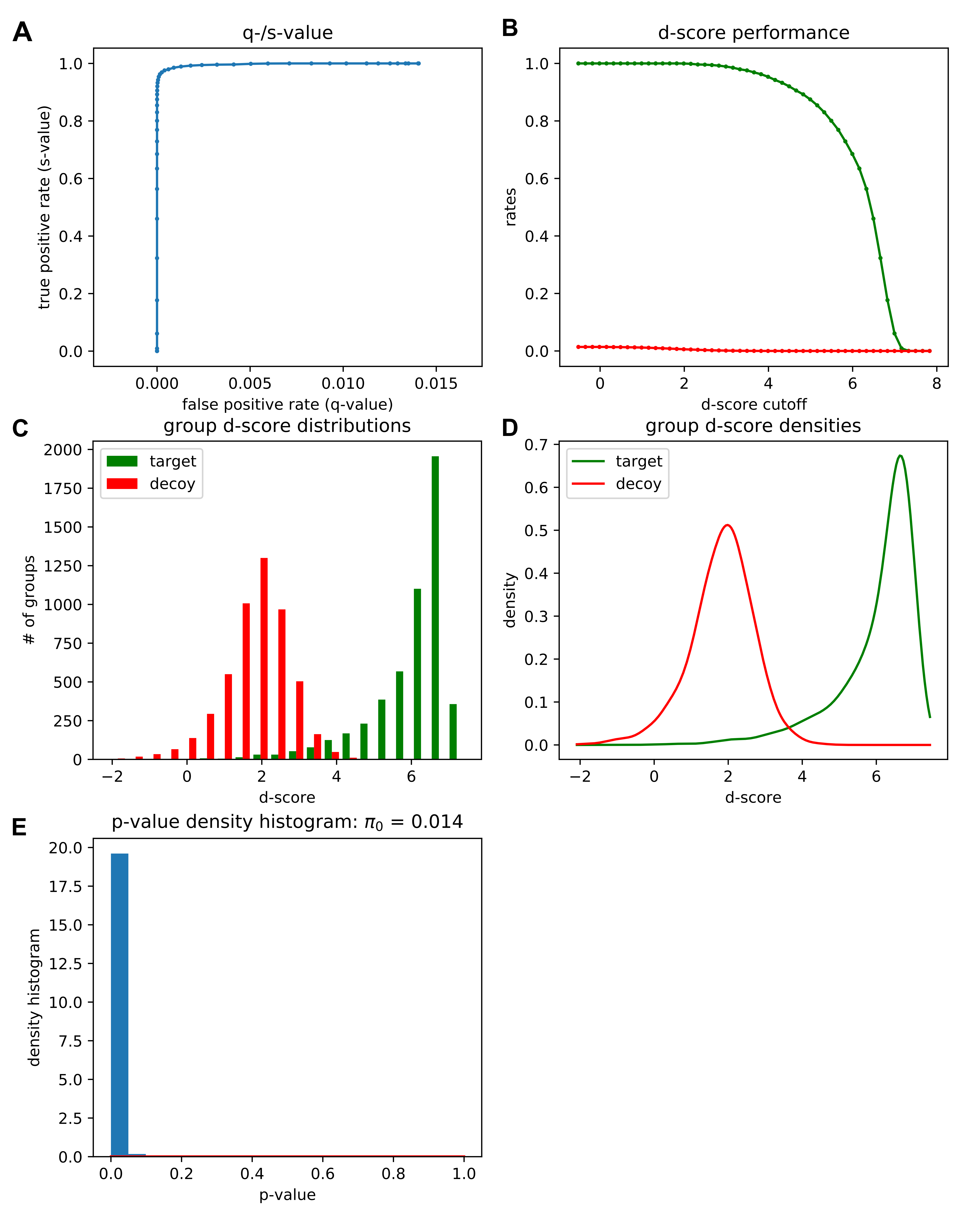

Supplement: giac005_Supplemental_Files [file giac005_supplemental_files.zip › Figure5_Supplementary.png]

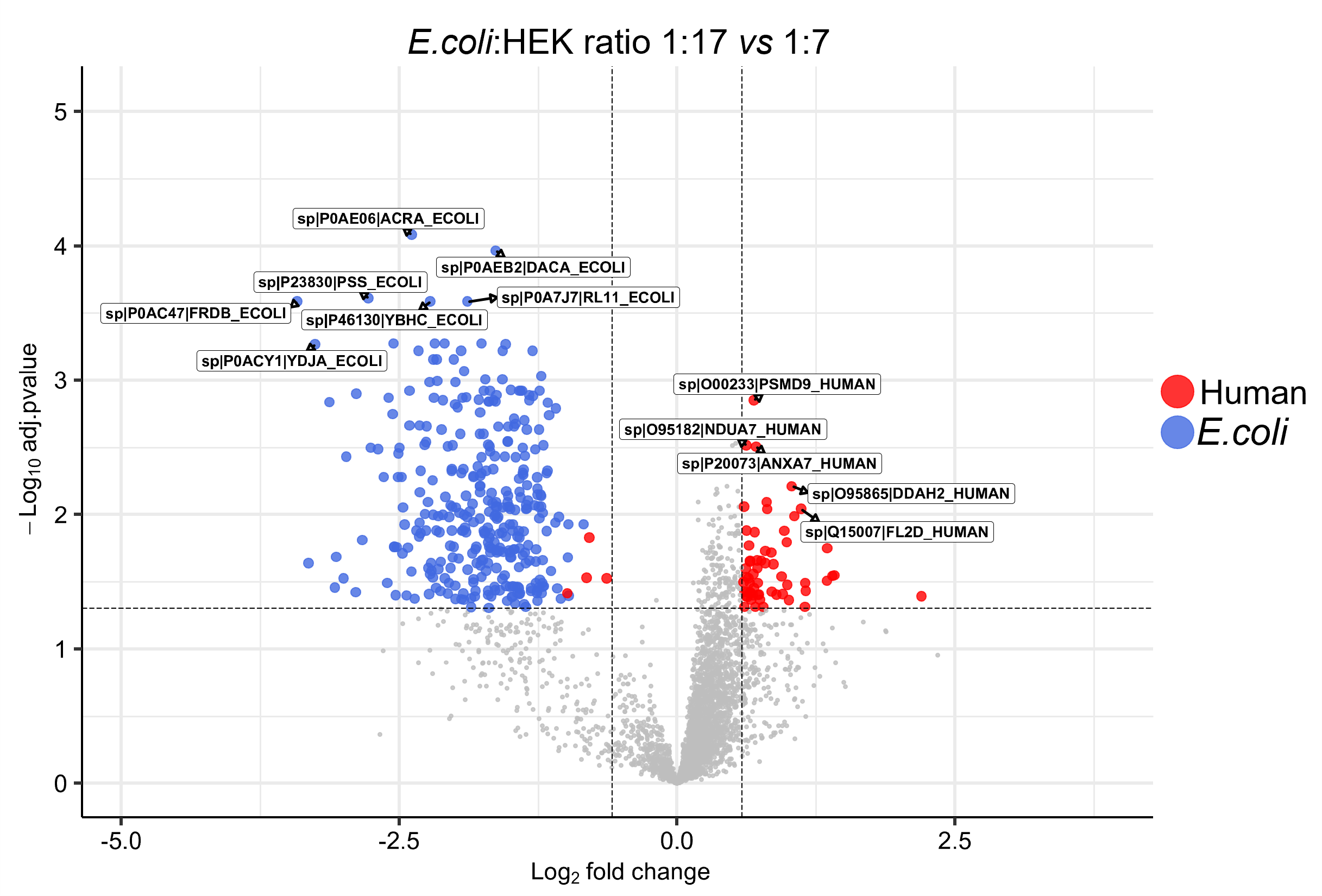

Supplement: giac005_Supplemental_Files [file giac005_supplemental_files.zip › Figure6_Supplementary.png]

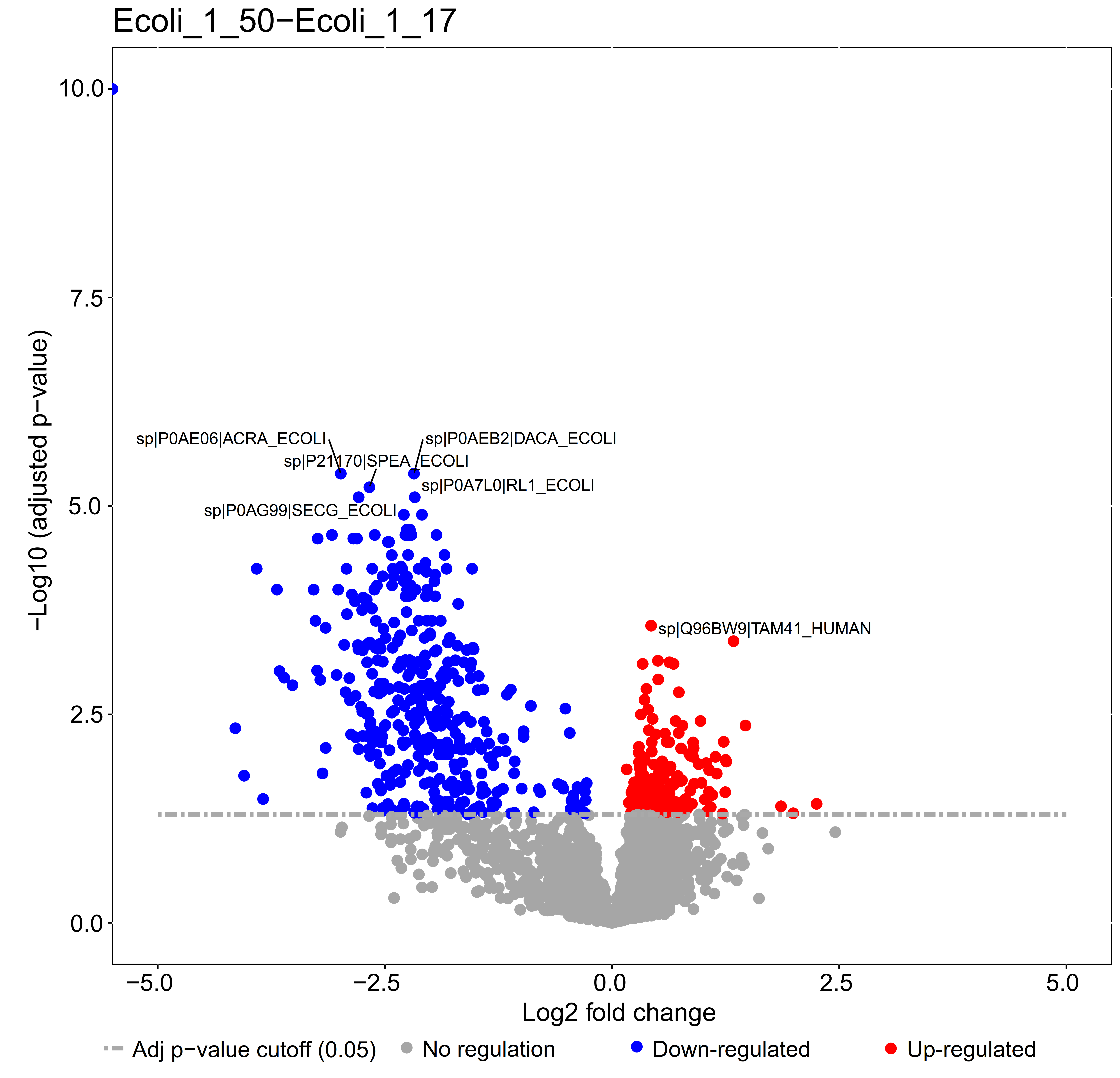

Supplement: giac005_Supplemental_Files [file giac005_supplemental_files.zip › Figure7_Supplementary.png]
