# Supplementary material for: Democratizing data-independent acquisition proteomics analysis on public cloud infrastructures via the Galaxy framework
Source: Gigascience. 2022 Feb 15;11:giac005. doi: 10.1093/gigascience/giac005 (PMC8848309; doi:10.1093/gigascience/giac005)
Supplement: giac005_Response_to_Reviewer_Comments_Revision_1 [file giac005_response_to_reviewer_comments_revision_1.pdf]

Author's Response To Reviewer Comments

Close

We sincerely thank the reviewers and editor for the helpful comments and suggestions. We agree with all the comments and adjusted the manuscript accordingly.

Reviewer #2:
Minor concerns

Comment 1:
“In their rebuttal the authors provided information on hardware requirement of some of the tools such as hundreds of GB of memory as well proposed to install galaxy from docker and then install the tools in their DIA processing workflow. I think this information is not only relevant for reviewer but more importantly for the users, which whish to use it in their own dedicated hardware. Description of the requirement as well installation procedure in supporting information or website would be very helpful.”

Answer: As rightfully pointed out by the reviewer we added the information about the docker installation of Galaxy and that some analysis steps require vast amounts of memory with the recommendation to use the European Galaxy instance.
“Galaxy can be installed as a docker container, however, a few steps in this workflow require up to hundreds of GB of memory, hence we recommend performing the DIA analysis on the European Galaxy server.”

Comment 2:
“The 400 GB is a very nice storage for the online platform for users, which information, I would include either in the paper as it allows users to plan the analysis.”

Answer: We added the information about the current resources for the PyProphet score tool as well as mentioned the possibility to extend the memory if required as well as added the respective location in GitHub.
“On the European Galaxy instance, the PyProphet score tool currently uses up to 400 GB of RAM, which can be further expanded up to one TB if required.”

Comment 3:
“On page 17 the RRID number: SCR_017410 (https://scicrunch.org/resolver/RRID:SCR_017410) seems to be a link for mass spectrometry imaging workbench and not for the DIA workflow described in the paper.”

Answer: We thank the reviewer for pointing this out. We adjusted the PRID number accordingly.
“RRID number: SCR_021862”

Comment 4:
“Using the link on Page 17 (section Availability of Supporting Data and Materials), I have tried to import history and workflow and run it, however the selection of the right files and whole procedure may be too complicated for a beginner. I would therefore suggest including a short tutorial on how to load a data from a history, a workflow how to select the input data to run a basic workflow.”

Answer: We fully agree with the reviewer´s suggestion and all three training materials contain detailed information on how to upload and proceed with data in Galaxy as well as how to perform the different analysis steps. Thus, we adjusted a sentence in the Accessibility and Training section of the manuscript emphasizing the step-by-step tutorial in the training.

“Each training contains step-by-step information about the data handling and processing as well as a brief introduction to the theory and principles of the respective analysis step.”

We sincerely thank the editor and the reviewer for the comments that helped to further improve the manuscript. We hope that the revised version of the manuscript will be deemed publishable.

Close
