# Supplementary material for: Democratizing data-independent acquisition proteomics analysis on public cloud infrastructures via the Galaxy framework
Source: Gigascience. 2022 Feb 15;11:giac005. doi: 10.1093/gigascience/giac005 (PMC8848309; doi:10.1093/gigascience/giac005)
Supplement: giac005_Reviewer_1_Report_Original_Submission — Paul Stewart -- 8/13/2021 Reviewed [file giac005_reviewer_1_report_original_submission.pdf]

Reviewer Report

Title: Democratizing Data-Independent Acquisition Proteomics Analysis on Public Cloud Infrastructures Via The Galaxy Framework

Version: Original Submission **Date:** 8/13/2021

Reviewer name: Paul Stewart

Reviewer Comments to Author:

Fahrner et al have produced a very nice manuscript and corresponding pipeline. They describe a collection of DIA tools in the Galaxy framework for reproducible and version-controlled data processing. These DIA tools are an excellent addition to the growing number of proteomics-centric tools already available in Galaxy. The reviewer could find no major revisions needed and therefore only requests a few minor revisions before this is ready for publication:
Please include page numbers in the revised manuscript to make referencing the text easier.
Page 6
OpenSwath and PyProphet are cited and are also used in the manuscript. Please cite one or two alternatives.
Please consider citing a tool the each time it is used in a new paragraph (e.g. MSstats).
There is heavy reliance on conjunctive adverbs (However, ...; Thus, ...) on this page and throughout the manuscript. These can make passages a bit hard to read. Please consider rephrasing.
Page 7
Why "so-called histories"? Aren't they simply "Histories"?
Page 14
'To decrease the analysis time of the semi-supervised learning, the merged OSW results can be first subsampled using the PyProphet subsample tool and subsequently scored using the PyProphet score tool. '
The reviewer is not familiar with this approach. Can you please give additional justification (maybe under methods?) or provide a citation that this is a reasonable approach?
Page 15
Please check your reference software and/or work with the journal to ensure that the web addresses are linked properly. For example, the reviewer tried copying the link "https://training.galaxyproject.org/training-%20material/topics/proteomics/tutorials/DIA_lib_OSW/tutorial.html" but a "%20" (or a space) is inserted into the URL after "training-" so the link as it appears did not work until this was removed. A less technically savy reader may think the links are broken and will not be able to access the materials.
Page 16
'We identified and quantified between 25.000 to 27.000 peptides ...'
Please be consistent with number formatting (25000 vs 25.000). Other values in the tables did not use this formatting. Please check with journal editor for convention.
Figures

Please be consistent with axes labels. Some are upper case and some are lower case.
Figure 2B
Please round R2 to 2 or 3 decimals.
Figure 3
Please change the red-green color scheme to a more color-blind friendly color scheme (e.g. red blue)

Level of Interest

Please indicate how interesting you found the manuscript: Choose an item.

Quality of Written English

Please indicate the quality of language in the manuscript: Choose an item.

Declaration of Competing Interests

Please complete a declaration of competing interests, considering the following questions:

- Have you in the past five years received reimbursements, fees, funding, or salary from an organisation that may in any way gain or lose financially from the publication of this manuscript, either now or in the future?
- Do you hold any stocks or shares in an organisation that may in any way gain or lose financially from the publication of this manuscript, either now or in the future?
- Do you hold or are you currently applying for any patents relating to the content of the manuscript?
- Have you received reimbursements, fees, funding, or salary from an organization that holds or has applied for patents relating to the content of the manuscript?
- Do you have any other financial competing interests?
- Do you have any non-financial competing interests in relation to this paper?

If you can answer no to all of the above, write 'I declare that I have no competing interests' below. If your reply is yes to any, please give details below.

I declare that I have no competing interests

I agree to the open peer review policy of the journal. I understand that my name will be included on my report to the authors and, if the manuscript is accepted for publication, my named report including any attachments I upload will be posted on the website along with the authors' responses. I agree for my report to be made available under an Open Access Creative Commons CC-BY license (http://creativecommons.org/licenses/by/4.0/). I understand that any comments which I do not wish to

be included in my named report can be included as confidential comments to the editors, which will not be published.

Choose an item.

To further support our reviewers, we have joined with Publons, where you can gain additional credit to further highlight your hard work (see: https://publons.com/journal/530/gigascience). On publication of this paper, your review will be automatically added to Publons, you can then choose whether or not to claim your Publons credit. I understand this statement.

Yes Choose an item.
