# Supplementary material for: Democratizing data-independent acquisition proteomics analysis on public cloud infrastructures via the Galaxy framework
Source: Gigascience. 2022 Feb 15;11:giac005. doi: 10.1093/gigascience/giac005 (PMC8848309; doi:10.1093/gigascience/giac005)
Supplement: giac005_Reviewer_2_Report_Original_Submission — Peter Horvatovich -- 9/5/2021 Reviewed [file giac005_reviewer_2_report_original_submission.pdf]

Reviewer Report

Title: Democratizing Data-Independent Acquisition Proteomics Analysis on Public Cloud Infrastructures Via The Galaxy Framework

Version: Original Submission **Date:** 9/5/2021

Reviewer name: Peter Horvatovich

Reviewer Comments to Author:

The article GIGA-D-21-00223 entitled "Democratizing Data-Independent Acquisition Proteomics Analysis on Public Cloud Infrastructures Via The Galaxy Framework" describes a targeted DIA LC-MS/MS processing workflow implemented in Galaxy framework. The paper describes the tools integrated in Galaxy environment and the workflows steps to process DIA LC-MS/MS data using targeted spectral library approach. The authors used a HEK cell lysate spiked with E.coli digest at various ratio and used these samples to generate DIA LC-MS/MS data on an Orbitrap QE+ with MS1 scans and 24 50% overlapping DIA windows between 400-1000 m/z in 4 replicates for each conditions. The implemented workflow contains the library generation from DDA data with MaxQuant processing, library cleaning and analysis of the DIA with OpenSWATH and statistical analysis using MSStat package in R. The authors present identification and quantification of proteins in the example data (differential analysis, volcano plot, CV plot).
The article has a potential interest to the proteomics community as it serves to promote the use of complex DIA data processing workflows in Galaxy web interface, which would otherwise require considerable programming skills and time to establish such workflow from the user. However, the authors should address some major and minor issues before I suggest the article to be accepted.
Major concerns:
1. The tools and the DIA processing workflows are implemented in Galaxy Europe, which is using for me unknown amount of resource in term of disk space and computational capacity (CPU, RAM). The authors should describe what is the limitations to use this online Galaxy server (maximum amount of upload, CPU time, is there any cost to use the service, limitation of RAM for the tools etc).
2. Some users do not want to use cloud-based services and public Galaxy server, but would wish to process their data (e.g. clinical sample from humans) on their own local computational closed infrastructure. For these users the authors should provide a tutorial, how to install Galaxy (just refer to Galaxy installation documentation) and how to get the tools from Galaxy toolshed and run their pipeline. Some users may have already a Galaxy server and getting additional tool may interfere, therefore I would strongly suggest creating a docker image where a single instance of Galaxy is installed with all necessary tools and include the raw data and settings in order to provide a clean workflow, that are sure to work.
3. I would also like to see data on actual runtime of the example dataset, specially focusing on FDR calculation as authors mention that a subsampling of the data is required for this.
4. I would also present peptide results as protein quantities are obtained after protein inference from multiple peptides, while the instrument is measuring peptides.

5. CV distribution of proteins in Figure 4a should be compared to other results from other dataset as it shows multimodal and large distribution, which seems to be independent from the spiking levels. This indicate some artifacts in the data.
6. The data is only submitted to time alignment using iRT peptides, but there is no normalization applied. The authors should check with box-plot/violin plot the individual distribution of peptides and proteins in each replicate and if necessary apply normalization to avoid "upregulated" human proteins. It would be also useful to color the dots in the volcano plot according to the species (human/E coli). The authors refer to displacement effects, which is not explained what it mean in the text (maybe ion suppression?).
7. Please provide the distribution of the missing values for each replicate as DIA should provide data with low percentage of missing (0) value.
Minors:
1. All figures and plots look like low resolution bitmap. Please provide high resolution plots preferable made from vector graphic.
2. Figure 2B, please restrict R2 numbers to 4 decimals.
3. Page 15, please explain what the contrast matrix is.
4. Page 15, I would replace "time consumption" to "required execution time"
5. The author mention in several place (e.g. page 19 and legend of table 2) that they have "developed tools" for DIA analysis. This is not true as they did not develop the original tools but integrated these tools in Galaxy environment in this study. Please correct this.
6. In figure 3 and supplementary figures 1-4 "Blot" is written, which I guess should be "Plot".
7. Page 21, Unix is mentioned as operating system, which I guess is not correct, but rather Linux is used. Please provide the distribution and version number.

Level of Interest

Please indicate how interesting you found the manuscript: Choose an item.

Quality of Written English

Please indicate the quality of language in the manuscript: Choose an item.

Declaration of Competing Interests

Please complete a declaration of competing interests, considering the following questions:

- Have you in the past five years received reimbursements, fees, funding, or salary from an organisation that may in any way gain or lose financially from the publication of this manuscript, either now or in the future?
- Do you hold any stocks or shares in an organisation that may in any way gain or lose financially from the publication of this manuscript, either now or in the future?

- Do you hold or are you currently applying for any patents relating to the content of the manuscript?
- Have you received reimbursements, fees, funding, or salary from an organization that holds or has applied for patents relating to the content of the manuscript?
- Do you have any other financial competing interests?
- Do you have any non-financial competing interests in relation to this paper?

If you can answer no to all of the above, write 'I declare that I have no competing interests' below. If your reply is yes to any, please give details below.

I declare that I have no competing interests.

I agree to the open peer review policy of the journal. I understand that my name will be included on my report to the authors and, if the manuscript is accepted for publication, my named report including any attachments I upload will be posted on the website along with the authors' responses. I agree for my report to be made available under an Open Access Creative Commons CC-BY license (http://creativecommons.org/licenses/by/4.0/). I understand that any comments which I do not wish to be included in my named report can be included as confidential comments to the editors, which will not be published.

Choose an item.

To further support our reviewers, we have joined with Publons, where you can gain additional credit to further highlight your hard work (see: https://publons.com/journal/530/gigascience). On publication of this paper, your review will be automatically added to Publons, you can then choose whether or not to claim your Publons credit. I understand this statement.

Yes Choose an item.
