# Supplementary material for: Democratizing data-independent acquisition proteomics analysis on public cloud infrastructures via the Galaxy framework
Source: Gigascience. 2022 Feb 15;11:giac005. doi: 10.1093/gigascience/giac005 (PMC8848309; doi:10.1093/gigascience/giac005)
Supplement: giac005_Reviewer_2_Report_Revision_1 — Peter Horvatovich -- 12/5/2021 Reviewed [file giac005_reviewer_2_report_revision_1.pdf]

Reviewer Report

Title: Democratizing Data-Independent Acquisition Proteomics Analysis on Public Cloud Infrastructures Via The Galaxy Framework

Version: Revision 1 **Date:** 12/5/2021

Reviewer name: Peter Horvatovich

Reviewer Comments to Author:

The reviewers addressed all comments and issues raised for their article entitled "Democratizing Data-Independent Acquisition Proteomics Analysis on Public Cloud Infrastructures Via The Galaxy Framework", which describes a targeted DIA LC-MS/MS processing workflow implemented in Galaxy framework. The authors addressed all emerged issues and comments adequately and I only have few very minor comments left:

1. In their rebuttal the authors provided information on hardware requirement of some of the tools such as hundreds of GB of memory as well proposed to install galaxy from docker and then install the tools in their DIA processing workflow. I think this information is not only relevant for reviewer but more importantly for the users, which whish to use it in their own dedicated hardware. Description of the requirement as well installation procedure in supporting information or website would be very helpful.
2. The 400 GB is a very nice storage for the online platform for users, which information, I would include either in the paper as it allows users to plan the analysis.
3. On page 17 the RRID number: SCR_017410 (https://scicrunch.org/resolver/RRID:SCR_017410) seems to be a link for mass spectrometry imaging workbench and not for the DIA workflow described in the paper.
4. Using the link on Page 17 (section Availability of Supporting Data and Materials), I have tried to import history and workflow and run it, however the selection of the right files and whole procedure may be too complicated for a beginner. I would therefore suggest including a short tutorial on how to load a data from a history, a workflow how to select the input data to run a basic workflow.

These issues are very minor and are only suggestion to the authors to improve the usability of the pipeline and therefore its inclusion do not require an additional round of review.

Level of Interest

Please indicate how interesting you found the manuscript: Choose an item.

Quality of Written English

Please indicate the quality of language in the manuscript: Choose an item.

Declaration of Competing Interests

Please complete a declaration of competing interests, considering the following questions:

- Have you in the past five years received reimbursements, fees, funding, or salary from an organisation that may in any way gain or lose financially from the publication of this manuscript, either now or in the future?
- Do you hold any stocks or shares in an organisation that may in any way gain or lose financially from the publication of this manuscript, either now or in the future?
- Do you hold or are you currently applying for any patents relating to the content of the manuscript?
- Have you received reimbursements, fees, funding, or salary from an organization that holds or has applied for patents relating to the content of the manuscript?
- Do you have any other financial competing interests?
- Do you have any non-financial competing interests in relation to this paper?

If you can answer no to all of the above, write 'I declare that I have no competing interests' below. If your reply is yes to any, please give details below.

No competing interest.

I agree to the open peer review policy of the journal. I understand that my name will be included on my report to the authors and, if the manuscript is accepted for publication, my named report including any attachments I upload will be posted on the website along with the authors' responses. I agree for my report to be made available under an Open Access Creative Commons CC-BY license (http://creativecommons.org/licenses/by/4.0/). I understand that any comments which I do not wish to be included in my named report can be included as confidential comments to the editors, which will not be published.

Choose an item.

To further support our reviewers, we have joined with Publons, where you can gain additional credit to further highlight your hard work (see: https://publons.com/journal/530/gigascience). On publication of this paper, your review will be automatically added to Publons, you can then choose whether or not to claim your Publons credit. I understand this statement.

Yes Choose an item.
